# Zona pellucida shear modulus, a possible novel non-invasive method to assist in embryo selection during in-vitro fertilization treatment

**DOI:** 10.1038/s41598-020-70739-y

**Published:** 2020-08-21

**Authors:** Elad Priel, Tsvia Priel, Irit Szaingurten-Solodkin, Tamar Wainstock, Yuval Perets, Atif Zeadna, Avi Harlev, Eitan Lunenfeld, Eliahu Levitas, Iris Har-Vardi

**Affiliations:** 1 Department of Mechanical Engineering, Shamoon College of Engineering, Beer-Sheva, 84100 Israel; 2 NRCN-Materials Engineering Department, Beer-Sheva, 84190 Israel; 3 Fertility and IVF Unit, Department of Obstetrics and Gynecology, Faculty of Health Sciences, Soroka University Medical Center and Ben-Gurion University of the Negev, POB 151, Beer Sheva, 84105 Israel; 4 Department of Public Health, Faculty of Health Sciences, Ben-Gurion University of the Negev, Beer Sheva, 84105 Israel; 5 Fertility and IVF Unit, Department of Obstetrics and Gynecology, Barzilai Medical Center, Ashkelon, Israel

**Keywords:** Computational models, Medical research, Mathematics and computing

## Abstract

The present study investigated the association between oocyte zona pellucida shear modulus (ZPSM) and implantation rate (IR). Ninety-three oocytes collected from 38 in-vitro fertilization patients who underwent intracytoplasmic sperm injection were included in this case–control study. The ZP was modeled as an isotropic compressible hyperelastic material with parameter $C_{10}$, which represents the ZPSM. Computational methodology was used to calculate the mechanical parameters that govern ZP deformation. Fifty-one developed embryos were transferred and divided into two groups—implanted and not implanted. Multivariate logistic regression analysis was performed to identify the association between ZPSM and IR while controlling for confounders. Maternal age and number of embryos per transfer were significantly associated with implantation. The IR of embryos characterized by $C_{10}$ values in the range of 0.20–0.40 kPa was 66.75%, while outside this range it was 6.70%. This range was significantly associated with implantation (*p* < 0.001). Geometric properties were not associated with implantation. Multivariate logistic regression analysis that controlled for relevant confounders indicated that this range was independently associated with implantation (adjusted OR 38.03, 95% confidence interval 4.67–309.36, *p* = 0.001). The present study suggests that ZPSM may improve the classic embryo selection process with the aim of increasing IR.

## Introduction

The zona pellucida (ZP) is a non-cellular layer of glycoproteins surrounding the oocyte^1^. Over the years, numerous studies have described an association between the physical state of a cell and its function or fate^2–5^. One of the most studied physical properties of oocytes is zona hardening, resulting from cortical granule release during fertilization^6^. This physical property, aimed to prevent polyspermy^6,7^, also occurs spontaneously in response to post-ovulatory aging, and results in poor fertilization rates^8–10^. Furthermore, spontaneous zona hardening is associated with exposure to in-vitro culture conditions^11,12^ that may consequently hinder the blastocyst’s potential to hatch the ZP. Although many studies have investigated animal oocytes or embryos, their observations link physical parameters to fertilization and developmental potential and not to implantation potential.

Several approaches have been described in the literature to measure the mechanic properties of the ZP^13^ by oocyte indentation^14,15^, compression^16^, or aspiration^17,18^. Based on these capabilities, two studies have used the physical inputs as selection criteria for embryos. Murayama et al. measured mouse ZP elasticity using a micro tactile sensor (MTS) system. They demonstrated specific changes in this elasticity during oocyte maturation, fertilization, and embryo development, and concluded that the MTS system can be applied to assisted reproductive technology (ART) to evaluate embryo quality^19^. Yanez et al. studied the mechanistic properties of human and mouse zygotes and reported that viscoelastic properties can serve as a predictor of blastocyst formation within hours after fertilization^18^. Although these two studies promoted the concept of utilizing oocyte-cell mechanics to improve embryo selection, to the best of our knowledge, the association of human ZP mechanical properties with implantation rate was not thoroughly investigated.

Although different methods are reported in the literature for measuring ZP mechanics, direct measurement of forces or pressures in clinical practice is not ethically acceptable. In order to circumvent this limitation, it is possible to apply computational methods, such as the Finite Element (FE) method, which solves the governing equations of momentum and provides an indirect estimation of the ZP mechanical response. The FE method has been used extensively in computational mechanics to investigate the mechanical behavior of biological materials at the organ^20^ and cellular levels^21,22^.

Physiological hatching of blastocysts and implantation are sequentially linked events. Hatching is controlled by a timely cooperative interplay of various cellular and molecular regulators, such as cytokines, growth factors, and proteases^23,24^. Thus, any disruption to this process may impair implantation. ZP thickness and its mechanical properties are other fundamental components in the hatching process. During this process, the expanded blastocyst applies mechanical pressure on the ZP causing an increase in its diameter coupled with a reduction in ZP thickness. This event is followed by the formation of a nick, which later expands and enables the embryo to hatch from the ZP and implant. Transfer of spontaneously hatched blastocysts results in a higher implantation rate compared to non-hatched ones^25,26^, suggesting that the chance of achieving pregnancy is influenced not only by the quality of the blastocyst, but also by the hatching capabilities.

In the present study, we hypothesized that oocyte ZP mechanics correlates with implantation rate. A computational methodology was used to calculate the mechanical parameters that govern ZP deformation and determine the ZP’s shear modulus (ZPSM), which is the ability to resist shape change. Using this methodology, we examined which values of SM could be associated with implantation.

## Materials and methods

### Study population

The study included 93 oocytes taken from 38 IVF patients that underwent intracytoplasmic sperm injection (ICSI). Fifty-one embryos were transferred, and the implantation rate was calculated. Only cycles with known implantation data where the number of gestational sacs matched the number of transferred embryos and embryos from cycles where no conception occurred were analyzed.

### Ovarian stimulation and luteal support

Two ovarian stimulation protocols were used: the gonadotropin-releasing hormone antagonist and the long gonadotropin-releasing hormone agonist protocols in combination with either human menopausal gonadotropin or recombinant follicle stimulating hormone. Final oocyte maturation was achieved by administering human chorionic gonadotropin—hCG (Ovitrelle; Merck-Serono, Germany) when at least two follicles of 16-mm in diameter were observed by ultrasound examination, and blood 17β-estradiol concentrations reached at least 500 pg/ml. Oocyte retrieval was performed 36–38 h after the administration of hCG. Embryos were transferred to the uterus using an abdominal ultrasound-guided technique. Patients were instructed to initiate luteal support using progesterone administered intravaginaly or intramusculary, combined with oral estrogen from the second day following oocyte retrieval until clinical pregnancy was determined. Clinical pregnancy was confirmed by the presence of gestational sac with fetal heartbeat by transvaginal ultrasound examination 4 weeks following oocyte retrieval.

### Oocyte retrieval and ICSI

Follicles were aspirated and the cumulus-oocyte complexes were washed in Global Total with HEPES medium (Life Global, Brussels, Belgium), oocytes were cultured in fertilization medium (Life Global, Brussels, Belgium) covered with mineral oil (Irvine Scientific, Santa Ana, CA, USA) for 3 h at 37 °C, 5.7% CO_2_, and 5% O_2_. Oocyte denudation was initiated by a 30-s incubation in 80 IU/mL of hyaluronidase (Irvine Scientific, Santa Ana, CA, USA), followed by washings in HEPES medium to remove enzyme residuals . Removal of cumulus cells from the oocyte was carried out by mechanical pipetting in Global Total medium containing HEPES. ICSI procedure was performed in the same medium at 400× magnification using a Nikon Eclipse Ti microscope.

### Oocyte geometrical parameters

ZP thickness and oocyte diameter were measured from digital images taken at three different orientations. These images, taken before the ICSI procedure, were analyzed using an in-house MATLAB code, which identified the boundary of the cytoplasmic core and the ZP. The boundary data were imported into a computer-aided design program as a point cloud, and specific 2D and 3D models of the oocyte were generated (Supplementary Fig. S1 online).

### Finite element analysis of ZP deformation

To simulate ZP deformation during the ICSI suction stage, the material model for the ZP and the boundary conditions must be defined. In this study, the ZP was modeled as an isotropic compressible hyper-elastic material, which can represent an elastic material undergoing large deformations.

A neo-Hookean strain energy density function with the standard decomposition into a volumetric and isochoric part^27^ yields:

1 $$\Psi \left( {I_{C} ,III_{C} } \right) = C_{10} \left( {I_{c}III_{C}^{-1/3} - 3} \right) + \frac{1}{{D_{1} }}\left( {III_{c}^{1/2} - 1} \right)^{2}$$

Here $I_{C} ,III_{C}$ are the first and third invariants of the right Cauchy deformation tensor (which are associated with the ZP actual deformation) and $C_{10} ,D_{1}$ are material constants which are related to the shear ($\mu$) and bulk modulus ($\kappa$) for the ZP, respectively $(C_{10} = \mu /2,D_{1} = \kappa /2)$. It should be noted that the value of $C_{10} ,D_{1}$ for each oocyte ZP is not known a priori.

During the ICSI fixation procedure, it was observed that the deformation of the ZP is localized to the area in contact with the pipette. Therefore, there is no deformation of the oocyte cytoplasmic core and inner cell material properties were taken to be similar to the ZP with no influence on computed results.

**Figure 1:**
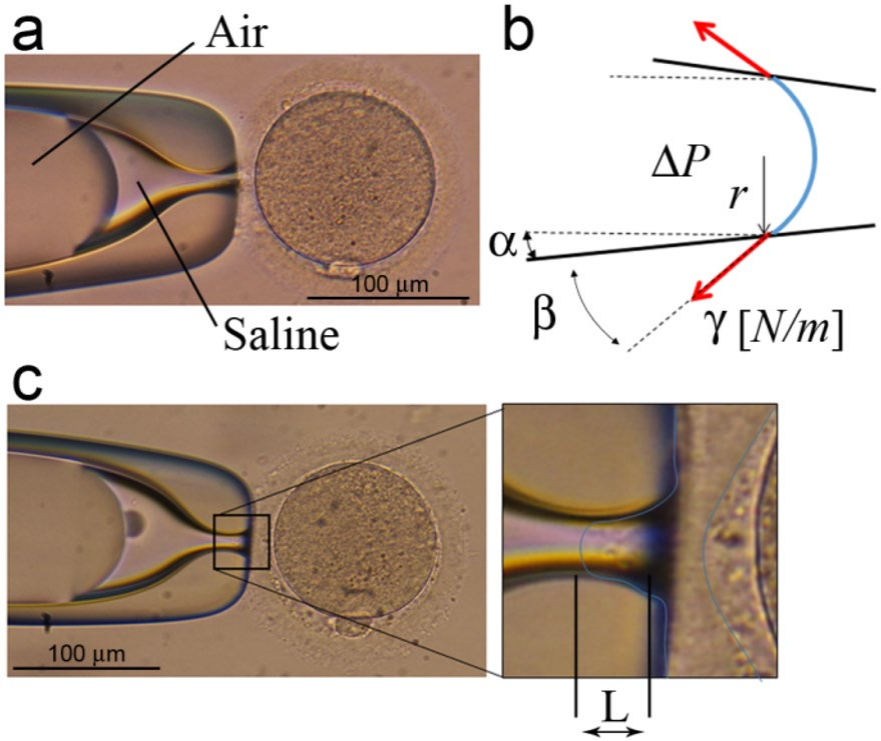
Analytical model for computing the suction pressure $\Delta P$**.** 2D image of the oocyte fixation stage showing the interface boundary between fluid and air (**A**). The different measurements required for estimation of suction pressure (**B**). 2D image of the oocyte fixation stage with a close-up look on the suction length L obtained for the estimated suction pressure $\Delta P$ (**C**).

The boundary conditions are defined by the suction pressure. Nevertheless, since no direct measurement of the suction pressure was possible in the clinical practice, the curvature of the air–fluid interface in the holding pipette was utilized to estimate this value. This stage was also used to measure ZP deformation due to the suction pressure (Fig. 1).

Analysis of the images was performed to measure the aspiration length L. Prior knowledge of surface tension and pipette geometry enabled calculation of the pressure gradient $\Delta P$ across the air–fluid interface, as shown in Eq. (2):

2 $$\Delta P = \frac{{2 \cdot \cos \left( {\alpha + \beta } \right)\gamma }}{r}$$

**Figure 2:**
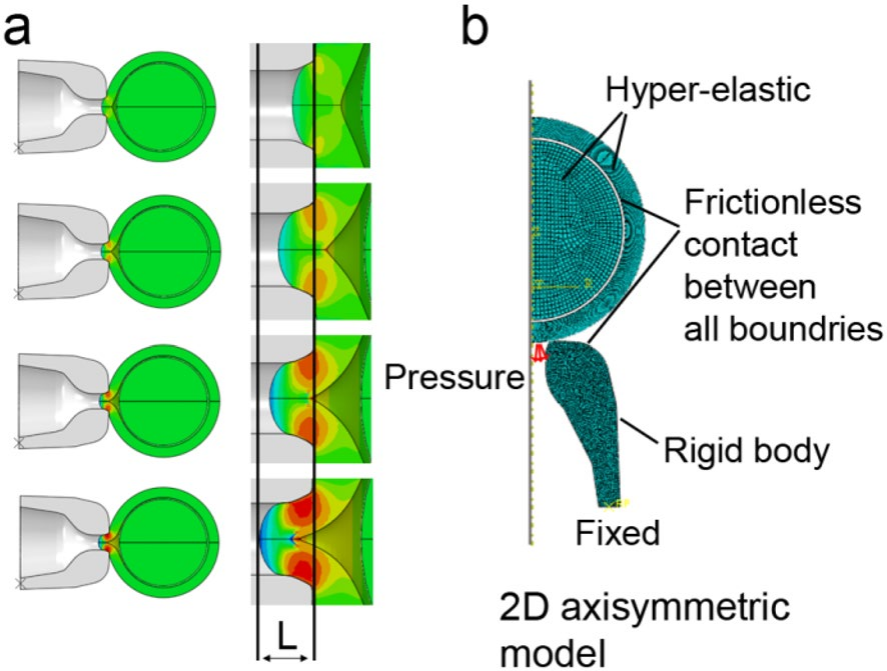
The finite element model and a representative deformation analysis of the ZP during aspiration. The computed suction length L is shown for different values of suction pressure (**A**). The finite element model developed for computing the oocyte deformation during the fixation stage (**B**).

The notation for the different parameters of Eq. (1) are provided in Fig. 1, with $\alpha$, the angle between the horizontal plane and the peptide, and $\beta$, the angle between the curvature of the air–fluid interface and the pipette wall. The local pipette cross-section radius is denoted by r and the value of air-fluid surface tension is denoted by $\gamma$. It should be noted that the value of r, $\alpha$, $\beta$ were measured via the image processing methodology for each specific oocyte. It was assumed that frictionless contact exists between the ZP and the pipette due to the liquid medium surrounding both the pipette and the oocyte. A preliminary computational analysis concluded that the incorporation of small friction coefficient values that differ from the frictionless assumption does not have significant influence on the computed results (Supplementary Fig. S2 online). To take any possible calculation errors into account, a sensitivity analysis was conducted which resulted in a maximal error of 5% in the aspiration pressure calculation using Eq. (2). This sensitivity analysis took into account errors in measurements of $r,\alpha ,\beta$, and the value used for the surface tension $\gamma$. An example of the computational model and the computed ZP deformation during the suction stage is shown in Fig. 2.

The computed aspiration length L was compared to the measured value. It is important to note that the material parameters $C_{10} ,D_{1}$ are a physical property of the ZP, which is not influenced by the applied suction pressure. For a given set of material parameters any change in suction pressure only influences the suction length L. A given oocyte measured under different values of suction pressure will result in different values of aspiration length L but will provide similar material parameters $C_{10} ,D_{1}$.

The computations revealed that the aspiration length L is sensitive to the value of $C_{10}$, but is not influenced by the value of the $D_{1}$ (which represents ZP resistance to volume change). As shown in Supplementary Fig. S3 online, the value of computed aspiration length L does not change for the different $D_{1}$ values used. As a result, only the value of SM can be accurately determined from analysis of the suction stage.

An iterative process was utilized and computations were conducted for varying values of $C_{10} ,D_{1}$ [see Eq. (1)] with the aim of minimalizing a target function given in Eq. (3):

3 $$Error[\% ] = \frac{{L_{M} \left( P \right) - L_{C} \left( P \right)}}{{L_{M} \left( P \right)}} \times 100$$

Here $L_{M} \left( P \right),L_{C} \left( P \right)$ are the pressure-dependent measured and computed aspiration lengths, respectively. The iterative process was concluded once the error between the computed and measured value of L was less than 1%. The values of SM and bulk modules ($C_{10} ,D_{1}$) at the final stage of the iteration were identified as mechanical parameters for the specific ZP examined.

### Validation of the computational model

Validation of the computational model used in this study requires direct measurements of pressure or force excreted on the ZP, which is not ethical in clinical practice. Nevertheless, partial validation was conducted using the following process; several of the ICSI procedures analyzed in this study had two sets of images taken during the fixation stage of the oocyte. In each set of images, the suction applied by the embryologist was different, resulting in a different value of aspiration length L. The first set of images was used for estimation of the ZP material parameters using the process outlined previously. Next, the computational model was used to predict the aspiration length L (using the same set of model parameters identified) for the second set of images. An example of this prediction process is shown in Supplementary Fig. S4 online.

### Embryo culture

Immediately after the ICSI procedure was performed, the injected oocytes were placed in culture slides (EmbryoSlide, Unisense FertiliTech, Aarhus, Denmark) containing 12 micro-wells, each filled with 25-µl droplets of a single step Global medium covered with mineral oil to prevent evaporation. The injected oocytes were incubated in a time-lapse incubator—an EmbryoScope system at 37 °C, 5.7% CO_2_, and 5% O_2_ (Unisense FertiliTech, Aarhus, Denmark).

### Embryo selection

Embryologists selecting embryos for transfer were blinded to oocyte geometrical properties (ZP thickness and diameter) and oocyte mechanics. Embryos were selected according to cleavage rate and morphological parameters^28^. Embryo transfer day was based on patient’s and physician’s decision.

Since embryos were transferred at different ages, the quality was assessed at 44 h post-ICSI using the conventional classification grading system (based on cell number, percentage of fragmentation, and blastomere symmetry)^29^. Based on this system, embryos were graded from 1 to 5 (1 indicating high-quality embryos; 5 indicating low-quality ones). None of the transferred embryos were graded 5.

### Data analysis and statistical methods

A case–control study was performed, in which embryos were divided into two groups based on whether they implanted (cases) or not (controls). Statistical analysis was performed using SPSS statistical software version 23 (SPSS Inc., Chicago, IL). Categorical variables are presented by the percentage of available observations. The χ^2^ test or Fisher’s exact test was used to compare between the study groups. Continuous variable data were presented using means and standard deviations, and compared between the groups using t-tests. Multivariate logistic regression analysis was performed to identify the association between ZPSM in a specific range and implantation rate. Variables were considered confounders based on the univariable comparison between the study groups (if associated with implantation and ZP range). The model included maternal age, number of retrieved oocytes and ZPSM in a specific range. Although the number of blastomeres and embryo grading at 44 h post-ICSI were not significantly associated with implantation, they were included in the model. The odds ratio (OR) and 95% confidence interval (95% CI) were computed, and all analysis were two-sided.

### Ethical approval

The clinical part of this study was conducted in a single Fertility and IVF Unit in a tertiary medical center during the years 2017–2019. The procedure was approved by Soroka university medical center review board (IRB reference 0041-14-SOR). All procedures performed in the study were in accordance with the ethical standards of the institution research committee and with the 1964 Declaration of Helsinki and its later amendments or comparable ethical standards. Patients were recruited to the study after obtaining their informed consent.

## Results

### Study population and implantation rates

The study included 93 oocytes that underwent an ICSI procedure, of which 51 of the developed embryos were transferred to 38 patients. Fourteen patients were conceived (36.84% clinical pregnancy rate) and 24 did not (63.16%). Two pregnancies consisted of two embryonic sacs. Patients were stratified to two groups based on treatment outcome (pregnant versus non pregnant). Patient's age was found to be significantly associated with implantation rate. The studied groups did not differ significantly from one another with regard to: levels of follicular stimulating hormone, luteinizing hormone and body mass index (Table 1). Estrogen level, number of retrieved oocytes, fertilization rate, embryo age at transfer, number of blastomeres and embryo grading at 44 h post-ICSI were also not associated with implantation rate (Table 1). However, the clinical pregnancy group had significantly lower mean number of embryos per transfer compared to the non-pregnant group (1.25 ± 0.44 versus 1.65 ± 0.48, respectively, p = 0.006).

**Table 1:** Patient characteristics of the study population.

Properties	Pregnant N = 14	Non-pregnant N = 24	*p*-value
Female age (year)	30.06 ± 5.98	34.37 ± 4.94	0.009
FSH (IU/L)	6.66 ± 2.57	6.33 ± 1.86	0.480
LH (IU/L)	4.75 ± 1.89	5.11 ± 6.12	0.472
Female BMI (kg/m^2^)	26.72 ± 6.62	25.46 ± 5.88	0.904
Number of transferred embryos	16	35	
Peak estrogen (pg/ml)	1,408.75 ± 362.95	1,423.54 ± 505.18	0.394
Oocytes retrieved	7.62 ± 2.18	7.0 ± 3.14	0.417
Fertilization rate (%)	72.76 ± 19.27	68.22 ± 22.42	0.514
Embryos per transfer	1.25 ± 0.44	1.65 ± 0.48	0.006
**Embryo age at transfer, % (N)**			
Day 2 + 3	28.9 (14)	70.2 (33)	0.403
Day 4 + 5	50 (2)	50 (2)	
Number of cells at day 2	4.12 ± 0.12	4.08 ± 0.19	0.159
Embryo grading at day 2	3.56 ± 0.20	3.68 ± 0.16	0.370

Values are presented as mean ± standard deviation unless indicated otherwise.

BMI, Body Mass Index; FSH, Follicular Stimulating Hormone; LH, Luteinizing Hormone.

### Geometrical and mechanical properties of the ZP and implantation rate

The geometrical properties of the oocyte/ZP in the study population indicated that non-implanted and implanted embryos were not significantly different from one another in mean ZP thickness, mean oocyte diameter and mean oocyte diameter divided by the ZP thickness. Mean ZPSM was found to be lower in the implanted group compared to the non-implanted group (Table 2) but the result was not statistically significant.

**Table 2:** Geometrical and mechanical properties of the oocyte/zona pellucida in the implanted versus non-implanted embryos.

Properties	Implanted N = 16	Non-implanted N = 35	p-value
ZP thickness (µm)	15.24 ± 2.57	15.20 ± 2.86	0.584
Oocyte diameter (µm)	159.39 ± 7.77	157.84 ± 7.92	0.838
Diameter/ZP thickness	10.72 ± 1.76	10.75 ± 2.2	0.455
ZP $C_{10}$ (kPa)	0.31 ± 0.80	0.35 ± 0.16	0.258

Values are presented as mean ± standard deviation. ZP, zona pellucida.

### Oocyte geometrical parameters ranges are not associated with implantation rate

Oocyte distribution according to the measured geometrical properties was examined in order to identify specific ranges associated with implantation rate (Supplementary Table S1 online).

None of these parameters ranges was significantly associated with implantation rate (ZP thickness, p = 0.594; oocyte diameter, p = 0.269; oocyte diameter divided by the ZP thickness, p = 0.848).

### Oocyte-specific ZPSM range is associated with implantation rate

**Figure 3:**
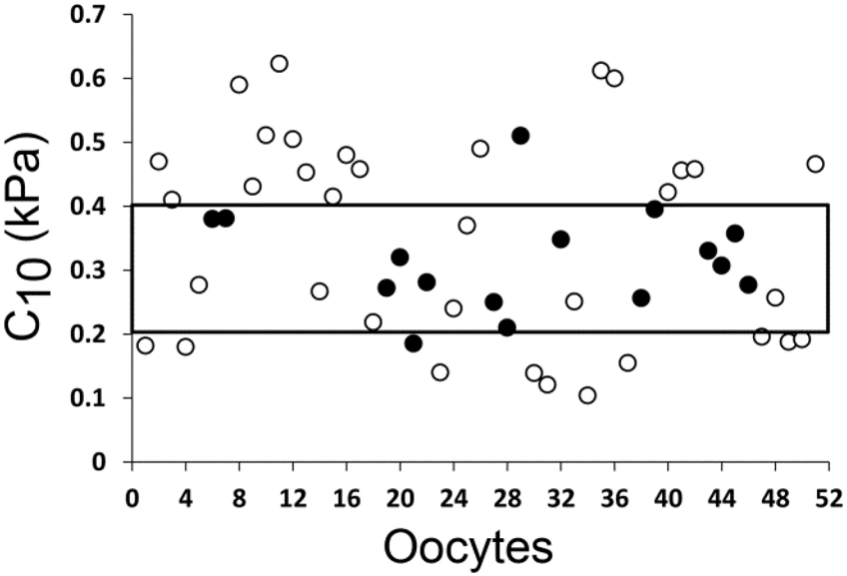
Oocyte distribution according to zone pellucida shear modulus. The zone pellucida shear modulus of 51 embryos was determined as described in “Materials and methods”. Black squares define a specific range with high implantation rate. Non-implanted and implanted embryos are marked by white and black circles, respectively.

To determine whether specific ranges of ZPSM can be associated with implantation, oocyte distribution according to this factor was evaluated (Table 3). Oocytes characterized by $C_{10}$ value in the ranges of 0.20–0.30 kPa and 0.30–0.40 kPa had the highest implantation rates (50% and 88.8%, respectively). Values of the $C_{10}$ below or above these ranges were associated with lower implantation rates. As shown in Fig. 3, 14 out of 21 embryos developed from oocytes characterized by $C_{10}$ values inside the range of 0.20–0.40 kPa were implanted (66.70%), while outside this range; only 2 out of 30 embryos were implanted (6.70%). The range was significantly associated with implantation rate (*p* < 0.001). The implanted embryos inside the range were not significantly different from the non-implanted ones inside the range with regard to embryo quality at 44 h post-ICSI reflected by the number of cells or embryo grading (*p* = 0.159 and *p* = 0.370, respectively).

**Table 3:** The distribution of the implanted embryos inside different shear modulus ranges.

$C_{10}$(kPa)	Oocytes N = 51	Implanted embryos N = 16	Implantation rate (%)
0.1–0.2	11	1	9
0.2–0.3	12	6	50
0.3–0.4	9	8	88.8
0.4–0.5	12	0	0
0.5–0.6	5	1	20
0.6–0.7	2	0	0

Multivariable logistic regression analysis, controlling for patient’s age, age plus mean number of transferred embryos, age plus mean number of blastomeres and mean embryo grading at 44 h post-ICSI, indicated that $C_{10}$ values in the range of 0.20–0.40 kPa were independently associated with implantation (Table 4).

**Table 4:** A multivariable analysis demonstrating the potential of an embryo characterized by $C_{10}$ values in the range of 0.200–0.400 kPa to implant.

	Adjusted OR	95% CI	p
Adjusted for age	22.09	3.92–124.45	< 0.001
**Adjusted for age and:**			
Number of transferred embryos	38.03	4.67–309.36	0.001
**Adjusted for age and:**			
Number of blastomeres	24.30	3.98–148.35	0.001
**Adjusted for age and:**			
Embryo grading at 44 h	21.68	3.84–122.34	< 0.001

### ZPSM is oocyte specific

**Figure 4:**
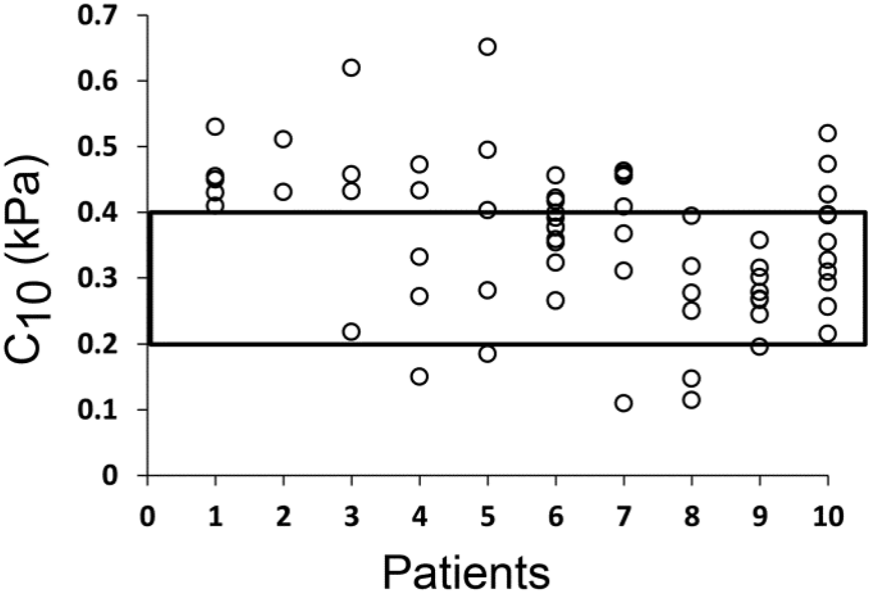
Intra-patient variations in the zona pellucida shear modulus of retrieved oocytes. The zone pellucida shear modulus (ZPSM) was determined as described in “Materials and methods”. Shown are $C_{10}$ values of oocytes, retrieved from 10 patients. The black square defines the $C_{10}$ range of 0.20–0.40 kPa. As shown, the distribution of the oocytes falls in different ranges, including outside and inside the range.

Analysis of oocyte ZPSM of 10 women, whom are part of the total 38 patients in the study, indicated that there is a variance in $C_{10}$ values between different oocytes for each woman, as shown in Fig. 4. Some of the oocytes fall inside the range of 0.20–0.40 kPa, while others fall outside this range.

## Discussion

The current study aimed to evaluate zona pellucida shear modulus as complementary non-invasive method to assist in embryo selection in order to increase implantation rates. The main finding of this study is that specific values of the ZPSM are associated with implantation in contrast to geometrical parameters such as, ZP thickness and oocyte diameter. Previous investigations on the thickness of the ZP have shown an association with pregnancy^30,31^. These studies examined ZP thickness at embryonic developmental stages, whereas the present study focused on oocyte measurements. To best of our knowledge this research is the first one to investigate ZP mechanics in a non-invasive specific technique.

In the current study, ZP mechanical properties were determined based on computed and measured aspiration length during routine ICSI procedures in a method similar to that used in the study of Khalilian et al.^32^. That study assumed that the ZP can be modeled using a linear elastic model. From a mechanical point of view, this model can be used for analysis of the oocyte suction stage, provided that there are small deformations and small strains of the ZP. As demonstrated in the present study, the ZP significantly deforms during the suction stage; thus, it was modeled as an isotropic compressible hyper-elastic material, which can represent an elastic material undergoing large deformations.

The hyper-elastic model utilized in the current study was also used to characterize the mechanical response of the mouse ZP. The study utilized Micro Electro Mechanical Mechanism System (MEMS) technology to directly measure forces acting on the ZP and identify, by FE analysis, the material model parameters^33^. The study reports $C_{10}$ values of 0.67, which are of the same order obtained in the current study for the human ZP.

A major effort in ART is directed towards developing an objective and measurable marker of embryo quality. The use of a time-lapse monitoring system^28,34–40^ has greatly improved the selection of viable high-quality embryos^36,38^. Using this system, morphokinetic parameters provide non-invasive criteria for embryo selection^41^. Still, some high-quality embryos fail to implant despite a receptive endometrium. Thus, additional parameters, which are not currently part of the embryo selection process, need to be defined and incorporated.

In this study, we hypothesized that ZP mechanics plays a vital role in implantation due to a link to embryo hatching. The embryo’s ability to complete the hatching process is mandatory for implantation to occur thus raising a possible association of the ZPSM and implantation rate. Even when high-quality embryos are selected for transfer, hatching and, subsequently, implantation may still be governed by ZP mechanics.

The observation of variances in the ZPSM between different oocytes of the same patient supports the conception that each oocyte is characterized by a specific ZPSM value. Some of the oocytes fall in the ZPSM range, that correlates with high implantation rates, while others fall outside of this range. We suggest that selection of embryos for transfer based not only on features of embryo quality but also on oocyte ZPSM values might improve implantation rates.

Recently, Yanez et al. utilized a linear viscoelastic material model to determine ZP mechanics and demonstrated that these mechanics can be used to assess human embryo viability at the zygote stage. According to their study, human oocyte mechanics can predict blastocyst formation. Furthermore, using a mouse model, embryos classified as viable, based on mechanics, were significantly more likely to result in a live birth compared with those classified as non-viable^18^. These findings and the present study support the hypothesis that the embryo’s developmental potential is determined by oocyte mechanics.

In the present study, patients with implanted embryos are younger than those with non-implanted embryos (Table 1), raising the assumption that endometrial receptivity may have an impact on embryo implantation potential and is better in younger patients. Endometrial receptivity is indeed fundamental for implantation and is tightly regulated by the coordinated action of estrogen and progesterone. In humans, no significant differences exist in estrogen receptor and progesterone receptor expression between young and aged endometrium^42^. In addition, changes of the endometrial line could be induced by exogenous sex steroid therapy, as performed during IVF treatment. Evaluation of endometrium function could be assessed by its receptiveness to good-quality embryos. In this regard, oocyte donation is the most appropriate model for such investigation. Young donors have good ovarian reserve and function, two prerequisite factors for good quality embryos. Soares et al*.* studied the outcomes of 3,089 oocyte donor cycles^43^. They concluded that there is no linear relationship between endometrium function and maternal age. With regards to the present study, we excluded the effect of maternal age by a multivariable logistic regression analysis (Table 4) that indicated $C_{10}$ values in the range of 0.20–0.40 kPa to be independently associated with implantation.

The methodological approach used in the present study has several advantages; First is its use of fresh human oocytes that circumvent any alterations in ZP mechanics^44^ and cortical granule exocytosis^45^ that may occur after fertilization or following frozen-thawed procedures. Second, the analysis of oocyte deformation applied during a routine ICSI procedure avoids any deviation from standard treatment. Third, its application may assist in embryo selection at early developmental stages, thus, avoiding extended time in culture.

The present study has several limitations; First, the estimation of the aspiration pressure during the suction stage is obtained from image-based calculations and not from direct pressure measurements. However, it is reasonable to assume that any error in aspiration pressure did not differ between oocytes and, therefore, did not influence the main finding that directly relates SM value and implantation potential. Second, the conclusion that the ZPSM is oocyte specific was based on limited numbers of oocytes that were analyzed. Thus, the research findings should be validated by larger prospective controlled studies. Although the present study does not offer immediate clinical application, its novelty relies in its concept which suggests that the potential of an embryo to implant may be determined by ZP mechanics.

In conclusion, this study describes a non-invasive method to measure ZPSM that may be associated with implantation potential. The authors suggest that embryo selection process in the future should combine embryo grading, based on standard criteria, as well as ZPSM value.

## Supplementary information

Supplementary Information 1.

## Data Availability

The manuscript includes all data that support the findings of the present study. Some supportive data is available in the supplementary material of the manuscript.
