# Supplementary material for: Zona pellucida shear modulus, a possible novel non-invasive method to assist in embryo selection during in-vitro fertilization treatment
Source: Sci Rep. 2020 Aug 21;10:14066. doi: 10.1038/s41598-020-70739-y (PMC7443135; doi:10.1038/s41598-020-70739-y)

Title: Zona pellucida shear modulus, a possible novel non-invasive method to assist in embryo selection during *in-vitro* fertilization treatment

Elad Priel[a,b], PhD, Tsvia Priel[c], PhD, Irit Szaingurten-Solodkin[c], PhD, Tamar Wainstock[d], PhD, Yuval Perets[c], Atif Zeadna[c], MD, Avi Harlev[e], MD, Eitan Lunenfeld[c], MD, Eliahu Levitas[c], MD, *Iris Har-Vardi[c], PhD

Supplementary Table

Supplementary Table S1. Oocyte distribution according to ranges of measurements of geometrical properties and implantation rates.

	**Oocytes N=51**	**Implanted embryos N=16**	**Implantation rate (%)**	**P-value**
**Thickness (μm):**				
10-15	26	8	30.8	
15-20	23	8	34.8	0.594
20-25	2	0	0	
**Diameter (μm):**				
140-150	8	3	37.5	
150-160	20	4	20	0.269
160-170	20	9	45	
170-180	3	0	0	
**Diameter/Thickness:**				
<8	2	0	0	
8-10	20	7	35	0.848
10-12	18	6	33.3	
12-14	6	2	33.3	
>14	5	1	20	

Legends for Supplementary Figures

Supplementary Fig. S1 online: The process of zona pellucida (ZP) material parameter estimation using oocyte images and computational analysis. Images of un-deformed (a) and deformed (b) oocytes were taken before and during intracytoplasmic sperm injection (ICSI), respectively. The deformed image illustrates fluid-air interface curvature in the pipette. Image analysis was performed to obtain oocyte ZP dimensions (c) and to identify the aspiration length L and fluid-air interface curvature (d). Computational model was established to simulate oocyte fixation during ICSI (e), f shows the computed pressure versus aspiration length L for different values of material model parameters. Comparisons were performed between observed and computed aspiration length L for determination of material model parameters for the specific oocyte ZP.

Supplementary Fig. S2 online: Influence of assumed friction value on computed aspiration length L. The figure demonstrates that the computed value of the aspiration length L is not influenced by the assumption of frictionless contact between the oocyte and the pipette. The dashed line represents the computed aspiration length L, as a function of applied suction pressure. The black circles represent a case in which a friction value of 0.1 is used.

Supplementary Fig. S3 online: Influence of bulk modulus D1 on the computed aspiration length. The figure demonstrates that the computed value of the aspiration length in the ICSI suction stage simulation is not sensitive to the value of the assumed bulk modulus value.

Supplementary Fig. S4 online: The prediction of aspiration length L following model parameter estimation. The computational model was validated using 2 sets of images obtained during intracytoplasmic sperm injection. One set of images was used to estimate the material parameter C_{10} for a specific oocyte (Black circle). Next, the computational model was used to predict the aspiration length L for various pressures applied. The solid line represents the predicted aspiration length L as a function of the applied pressure. As shown, the measured aspiration length for image set 2 (Black Square) is in accordance with the predicted aspiration length. The maximal error in the predicted aspiration length L compared to the observed value was 7%.

Supplementary Figures

Supplementary Figure S1

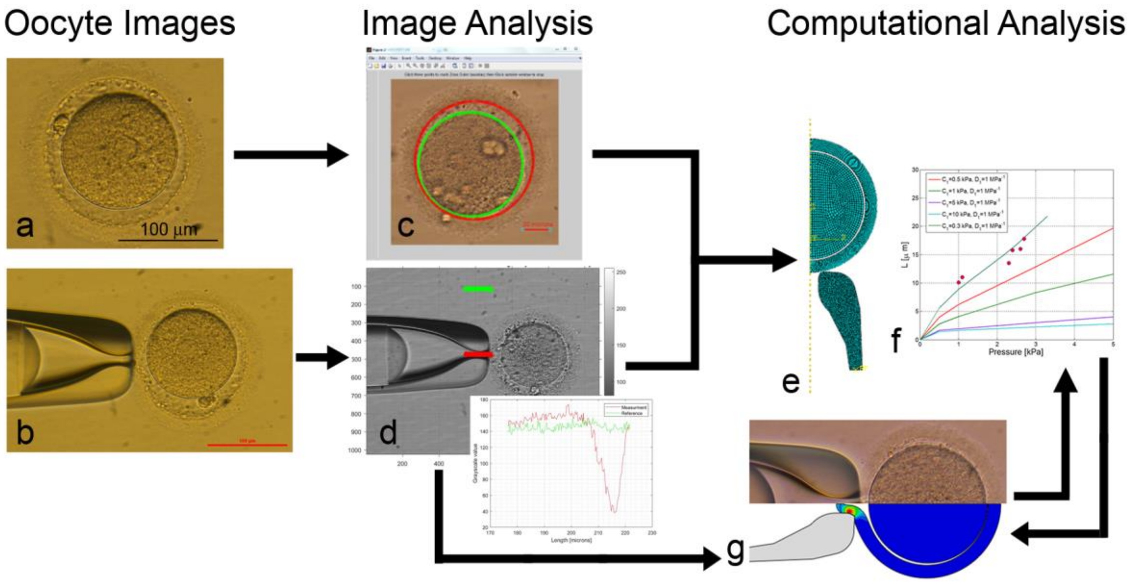

Supplementary Figure S2

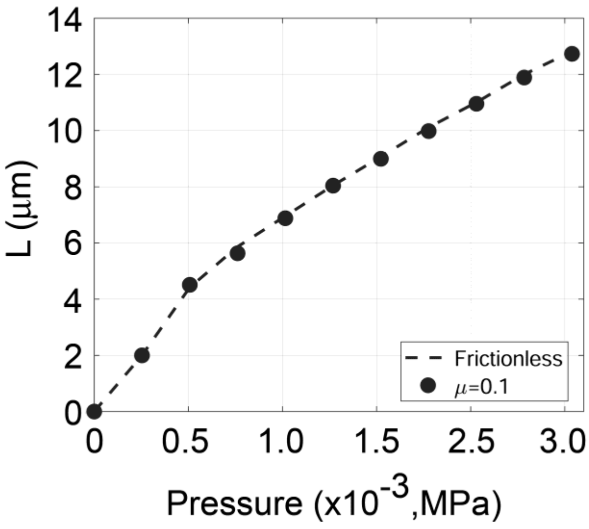

Supplementary Figure S3

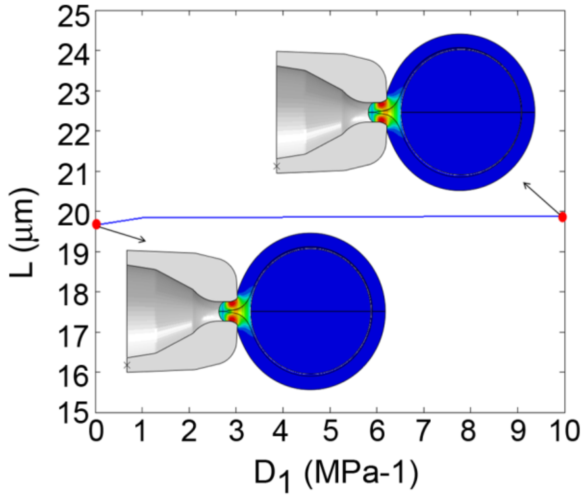

Supplementary Figure S4

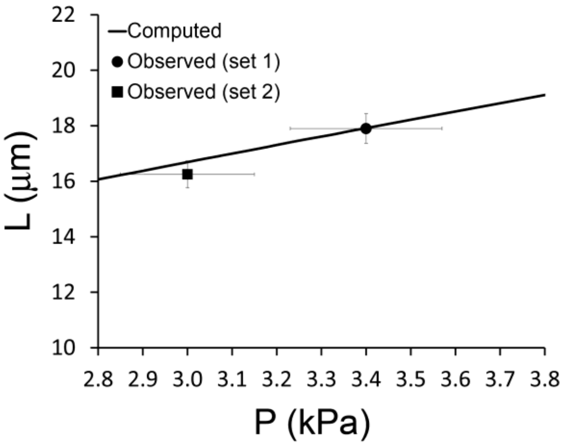

Supplement: Supplementary file 1 — Supplementary Information 1. [file 41598_2020_70739_MOESM1_ESM.pdf]
